# Antineutrophil cytoplasmic antibodies (ANCA)-positive patient with infective endocarditis and chronic hepatitis B virus: a case report and review of the literature 

**DOI:** 10.1186/s13256-020-02373-1

**Published:** 2020-07-06

**Authors:** Qian Zhang, Baoxian Shi, Hongbing Zeng

**Affiliations:** 1 Division of Nephrology, Department of Internal Medicine, Tongji Hospital, Tongji Medical College, Huazhong University of Science and Technology, Wuhan, Hubei People’s Republic of China; 2 Department of Chemistry and Environmental Engineering, Wuhan Polytechnic University, Wuhan, Hubei People’s Republic of China

**Keywords:** Case report, Antineutrophil cytoplasmic antibodies, Infective endocarditis, Hepatitis B virus infection

## Abstract

**Background:**

Antineutrophil cytoplasmic antibodies comprise a family of autoantibodies that are often used as biomarkers for certain forms of small-vessel vasculitis; however, chronic infections tend to induce the production of antineutrophil cytoplasmic antibodies. Infective endocarditis and hepatitis B virus infection have been reported to exhibit antineutrophil cytoplasmic antibody positivity and to mimic antineutrophil cytoplasmic antibody–associated vasculitis, which may lead to misdiagnosis and inappropriate treatment.

**Case presentation:**

We report a case of a 46-year-old Han Chinese man with untreated chronic hepatitis B virus infection who featured proteinase-3 antineutrophil cytoplasmic antibody positivity while hospitalized with infective endocarditis. Cardiac ultrasound echocardiography disclosed mitral and aortic regurgitation with vegetation. On the 15th hospital day, the patient underwent mitral and aortic valve replacement and was then treated with antibiotics for more than 1 month. On the 57th hospital day, the patient was discharged. His urinary abnormalities and renal function were gradually recovering. Four months after being discharged, his proteinase-3 antineutrophil cytoplasmic antibody levels had returned to the normal range.

**Conclusions:**

The findings in this study update and expand current understanding of antineutrophil cytoplasmic antibody positivity in patients with both infective endocarditis and hepatitis B virus. Treatment (including surgery, antibiotics, corticosteroids and/or cyclophosphamide, antiviral agents, and even plasma exchange) is challenging when several diseases are combined. Renal biopsy is suggested if the patient’s condition allows. Antineutrophil cytoplasmic antibody testing should be repeated after therapy, because some cases might require more aggressive treatment.

## Background

Antineutrophil cytoplasmic antibodies (ANCAs) comprise a family of autoantibodies that react with proteins predominantly expressed in cytoplasmic granules of polymorphonuclear neutrophils [1]. Indirect immunofluorescence assays can distinguish ANCAs with cytoplasmic (c-ANCA) or perinuclear (p-ANCA) staining patterns; autoantibodies with specificity for myeloperoxidase, referred to as “MPO-ANCA,” and those against proteinase-3, referred to as “PR3-ANCA,” can be further characterized by enzyme-linked immunosorbent assay [2]. The c-ANCA pattern is, in most cases, caused by antibodies to PR3, and MPO can be responsible for the p-ANCA pattern [1]. The presence of these autoantibodies is an important diagnostic marker for small-vessel vasculitic syndromes (i.e., granulomatosis with polyangiitis, microscopic polyangiitis, eosinophilic granulomatosis, and polyangiitis), which are commonly referred to as “antineutrophil cytoplasmic antibody–associated vasculitis” (AAV) [3]. However, ANCA positivity can be seen in a variety of infectious diseases and in a variety of autoimmune diseases, including infective endocarditis (IE), systemic lupus erythematosus (SLE), rheumatoid arthritis, inflammatory bowel disease, hepatitis B or C virus (HBV or HCV, respectively) infection, and human immunodeficiency virus (HIV) infection [4]. Because AAV and infectious diseases may present similarly, ANCA positivity must be carefully interpreted [5]. Moreover, patients with either IE or HBV can present with ANCA positivity, leading to more difficulties in diagnosis and treatment. This case report describes a 46-year-old man with chronically untreated HBV infection who was admitted to our hospital with IE and was found to be c-ANCA-positive. We also summarize the literature of previously published cohort cases concerning ANCA induction in IE and HBV infection.

## Case presentation

In July 2017, a 46-year-old man of Han Chinese ethnicity was referred from a local community hospital with complaints of fever of 2 weeks’ duration, along with hematuria, proteinuria, and rapidly deteriorating renal function. He was being treated with intravenous antibiotics and expectant treatment for his kidneys without any improvement. The patient had a 5-year history of hypertension without treatment (peak blood pressure 145/110 mmHg). In February 2017, he was diagnosed with pancreatitis, and he recovered after treatment. He denied any history of diabetes, alcohol intake, intravenous drug abuse, and smoking. He had no history of familial disease and no known environmental exposure. He was a farmer, was married, and had a daughter. He had not undergone any examination for infectious diseases, including HBV, HCV, or HIV.

On admission to our hospital, his mental status was normal. His body temperature was 36.9 °C, pulse rate was 92 beats/min and regular, respiratory rate was 18 breaths/min, and blood pressure was 116/78 mmHg. His physical examination revealed a systolic murmur (Levine classification 3/6) in the apex area and a diastolic murmur (Levine classification 2/6) in the aortic area. The result of his neurological examination was normal. A series of laboratory tests was performed after admission.

**Fig. 1:**
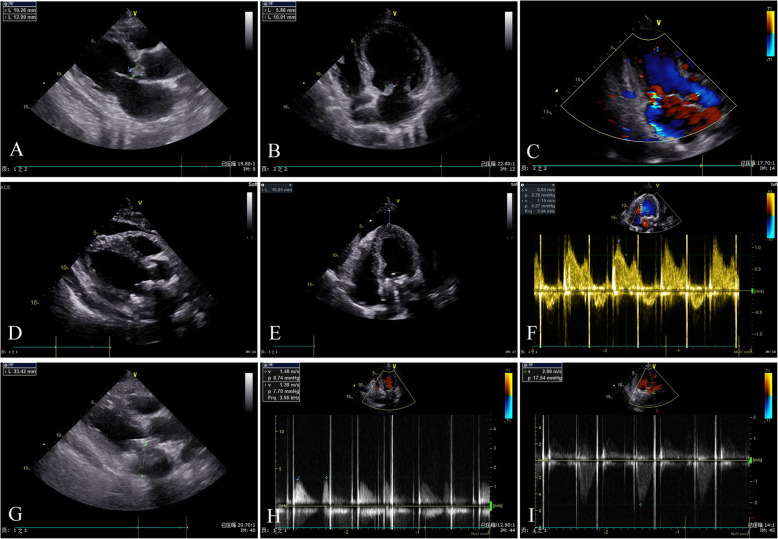
The echocardiogram findings of our patient with antineutrophil cytoplasmic antibody–positive infective endocarditis. **a**–**c** Echocardiograms reveal aortic and mitral valve vegetations (13 × 10 mm, 11 × 6 mm) and regurgitation before surgery. **d**–**f** Echocardiograms reveal normal function in prosthetic valves 14 days after aortic and mitral valve replacement and the color Doppler blood flow spectrum. **g**–**i** Echocardiograms reveal the normal function in prosthetic valves 42 days after aortic and mitral valve replacement and the color Doppler blood flow spectrum

**Fig. 2:**
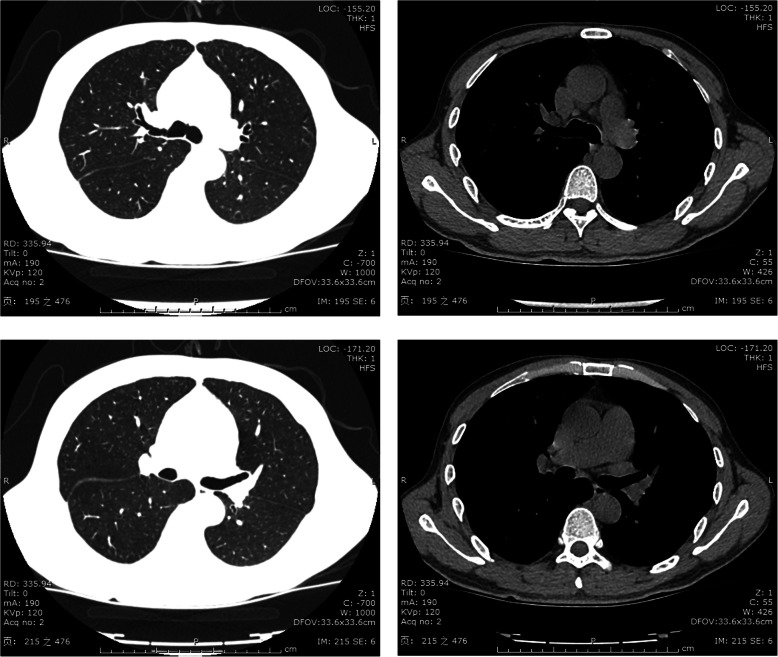
Chest computed tomographic scans of our patient with antineutrophil cytoplasmic antibody–positive infective endocarditis

On the day of admission, laboratory results indicated 1+ proteinuria (1.28 g/24 h), 3+ urine occult blood with 2959.9 red blood cells per high-power field, a white blood cell count of 6.22 × 10^9^/L, hemoglobin of 81 g/L, albumin level of 32.3 g/L, globulin level of 48.7 g/L, serum creatinine level of 196 μmol/L, and blood urea nitrogen level of 7.7 mmol/L. His hepatitis B surface antigen, hepatitis B e-antigen (HBeAg), and hepatitis B core antibodies were positive. His DNA level of HBV was 1.38 × 10^4^ IU/ml. The findings for rheumatoid factor, HCV antibody, and HIV were negative. The level of MPO-ANCA was normal, whereas that of PR3-ANCA was 170.4 relative units/ml (normal range, < 20). Blood cultures were obtained three times, and examination revealed the presence of *Abiotrophia defectiva*. Ultrasound cardiac examination revealed aortic and mitral valve vegetation (13 × 10 mm, 11 × 6 mm). Moderate regurgitation and turbulence could be seen in the diastole of the aortic valve orifice and the systole of the left atrial side of the mitral valve orifice (Fig. 1a–c). A chest computed tomographic scan showed no obvious pulmonary infection (Fig. 2).

Given the clinical information provided, including the results of the blood culture and ultrasound cardiac examination, IE was identified. However, it was difficult to determinate whether HBV or more acute IE was the main cause of this patient’s ANCA positivity. Due to the patient’s rapidly deteriorating renal function, he was supposed to undergo kidney biopsy to clarify the type of glomerulonephritis (GN), but the results of echocardiography stopped us from performing a biopsy. The patient’s echocardiogram revealed mitral and aortic valve vegetation (11 × 6 mm, 13 × 10 mm) and regurgitation. Doctors in the cardiothoracic surgery department suggested valve replacement surgery be considered to avoid systemic bacterial emboli. Severe regurgitation and heart failure due to valvular destruction was another consideration. Because of these complex conditions, the patient’s diagnosis was a challenge.

**Fig. 3:**
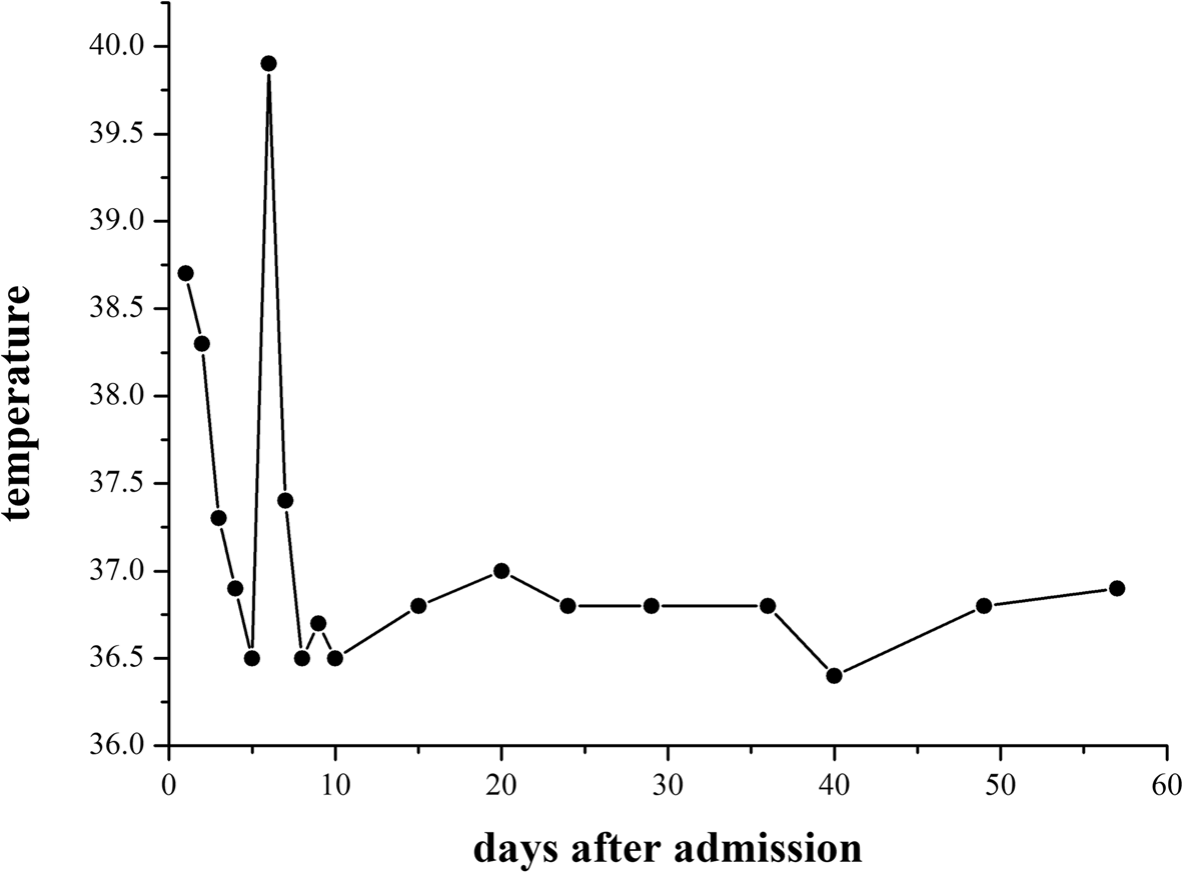
The curve of the patient’s daily temperature after admission

**Fig. 4:**
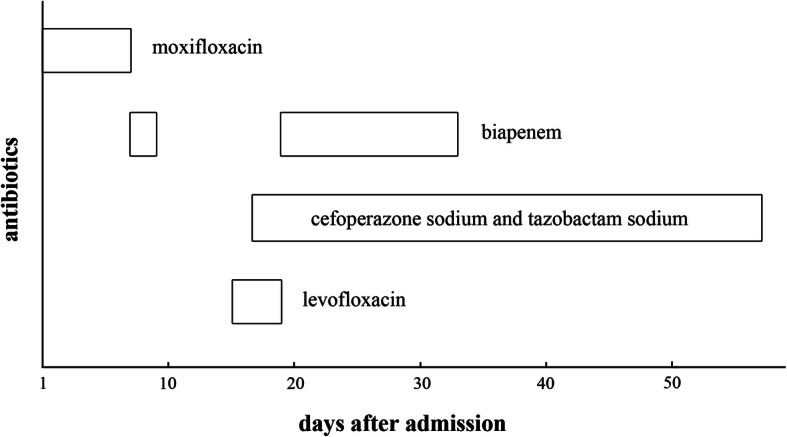
The course and doses of antibiotics after admission (moxifloxacin, 400 mg intravenous drip once daily; biapenem, 300 mg intravenous drip every 8 h; cefoperazone sodium and tazobactam sodium, 2000 mg intravenous drip every 12 h; levofloxacin, 300 mg intravenous drip once daily)

**Fig. 5:**
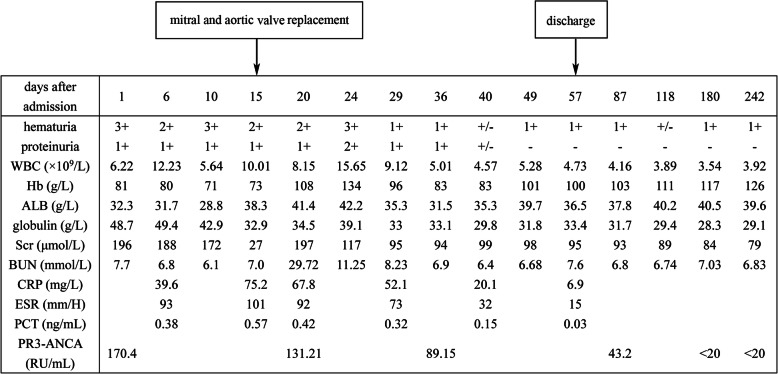
The clinical course of our patient with antineutrophil cytoplasmic antibody–positive infective endocarditis

When the patient was admitted to our hospital, he had a fever the first night. He was treated with antibiotics because blood culture results indicated *A. defectiva* infection. The patient’s temperature decreased gradually (Fig. 3). Meanwhile, he was given other medications, including niferex (150 mg once daily by mouth), folic acid (5 mg three times daily by mouth), and medicinal charcoal tablets (1.2 g three times daily by mouth). On the 15th hospital day, the patient underwent mitral and aortic valve replacement and was then treated with warfarin (2.5 mg once daily by mouth) and antibiotics for more than 1 month (Fig. 4). On the 57th hospital day, the patient was discharged. His urinary abnormalities and renal function were gradually recovering. Four months after being discharged, his PR3-ANCA levels had returned to the normal range. The patient’s whole clinical course can be seen in Fig. 5.

## Discussion

This case report describes a 46-year-old man who featured PR3-ANCA positivity with fever, along with hematuria, proteinuria, and rapidly deteriorating renal function. It was easy to imagine the diagnosis of vasculitis. However, the patient had both untreated chronic HBV infection and IE, and ANCA positivity could be present in both diseases. Because more than one factor was affecting the ANCA positivity in this patient, it was necessary to identify the main factor to determine the appropriate treatment.

ANCA is a biomarker for certain forms of small-vessel vasculitis, but specificity is limited, especially when detected with indirect immunofluorescence [1]. Apart from vasculitis, ANCA with different specificities occurs in a wide range of conditions requiring very different actions, such as IE, SLE, rheumatoid arthritis, inflammatory bowel disease, HBV or HCV infection, and HIV infection [1, 4]. Diagnosis may be hampered by nonspecific symptoms and can be made more difficult by association with autoantibodies. Therefore, a careful diagnostic workup is warranted.

IE, a microbial infection of the endocardial surface of the heart, is classified as “acute” or “subacute chronic” on the basis of the tempo and severity of the clinical presentation and the progression of the untreated disease [6]. The incidence of IE in the general population ranges from approximately 2 to 7.9 per 100,000 individuals per year and has a short-term mortality rate of 10% to 30% [7–9]. Bacterial colonization of the cardiac valves may lead to local meltdown with abrupt-onset cardiac failure as well as systemic spread of infectious particles covered with antibodies and complement. The latter may stimulate multiple immunological abnormalities and develop into a subacute inflammatory condition mimicking systemic vasculitis [1, 10]. The treatment is urgent administration of combinations of antibiotics; sometimes surgery is necessary to restore the integrity of the valves [1]. However, the development of ANCA-mediated disease during the course of IE raises the difficulty of determining the specific treatment. Treatment with immunosuppressive medications may increase the risk of septic death [11]. Also, treatment of the endocarditis with appropriate antibiotics usually leads to abolition of the immunological abnormalities and their clinical manifestations [4]. Controversy has been generated regarding appropriate treatment in ANCA-positive patients with IE. Our case report describes a cure of an ANCA-positive patient with IE who had appropriate antibiotic treatment and valve replacement surgery. The patient started with fever, hematuria, and proteinuria, and then his renal function gradually deteriorated. Examination of antibodies showed c-ANCA and PR3-ANCA. It has been reported that kidney disease is a common manifestation of IE, involving nearly 40% to 50% of patients [12]. Endocarditis-associated GN can show significant variability in histopathologic appearance, including pauci-immune GN, postinfective GN, or subendothelial membranoproliferative GN [13, 14]. Because of this variability, a patient’s renal disease could be misdiagnosed as vasculitis rather than IE-mediated GN, and the differentiation can be very challenging. For this reason, the patient was supposed to undergo kidney biopsy, but the results of echocardiography stopped us from performing it. The patient’s echocardiogram revealed mitral and aortic valve vegetation (11 × 6 mm, 13 × 10 mm) and regurgitation. Blood culture results indicated *A. defectiva* infection. Doctors from the cardiothoracic surgery department suggested that valve replacement surgery as soon as possible should be considered to avoid systemic bacterial emboli due to the size of the vegetation and fast aortic blood flow as well as severe regurgitation and heart failure due to valve destruction. Therefore, surgery was performed, and two artificial valves were used. We were unable to perform a kidney biopsy, because anticoagulation drugs were used after the valve replacement. So, the type of GN was not clear in this case. However, by 6 months after surgery and with appropriate antibiotic treatment, the urinary abnormalities had improved, and the patient’s PR3-ANCA level was normal. Therefore, it was not necessary to provide treatment with long-term immunosuppression.

Another ANCA-associated infection present in our patient was HBV infection. Because ANCA induction is more common in chronic infections [15], it was difficult to determinate whether HBV or more acute IE was the main cause of this patient’s ANCA positivity. Moreover, it is important to determine whether HBV stimulates ANCA formation without triggering vasculitis or other immune-related manifestations [16]. Vasculitis is one type of extrahepatic manifestation of HBV infection, and polyarteritis nodosa (PAN) is the most common vasculitis associated with acute or chronic HBV infection [17]. The frequency of HBV-associated PAN has been reported to range from 17.4% to 48.8% and has come down over the years because of screening of blood products before transfusion and increasing vaccination [18–20]. HBV-related PAN is an acute disease, and patients often present with gastrointestinal tract involvement, vascular nephropathy, malignant hypertension, and orchitis [18, 21]. Our patient had no clinical manifestations other than renal damage. HBV is considered to contribute to vasculitis development through at least two mechanisms: viral replication induces direct damage to the blood vessel wall leading to viral arteritis, and vascular damage could be associated with immune-complex reactions [16, 22]. The DNA level of HBV in our patient was 1.38 × 10^4^ IU/ml; his liver function was normal; and he had no evidence of liver cirrhosis. Therefore, antiviral therapy was not recommended according to the guideline of prevention and treatment for chronic HBV suggested by the Chinese Society of Hepatology, the Chinese Medical Association, the Chinese Society of Infectious Diseases, and the Chinese Medical Association [23]. The patient’s condition was relieved, and his level of PR3-ANCA returned to normal by means of surgery and anti-infective treatment, which further proved that the ANCA positivity was associated with IE rather than HBV.

To date, over 70 ANCA-positive patients with IE have been reported, the majority of which were described in single-case reports [5]. In order to further investigate the prevalence and characteristics of ANCA in patients with IE and patients with HBV, we performed a literature search of previously published cohort cases via PubMed and Medline using the keywords “infective endocarditis,” “IE,” “ANCA,” “antineutrophil cytoplasmic antibodies,” “hepatitis B,” and “HBV.” We list the results of our literature search in Table 1, describing the clinical features of IE and HBV in cases with ANCA positivity [3, 14, 16, 18, 24–29]. The results showed that the prevalence of ANCA positivity was higher in patients with IE than in patients with HBV (26.64% vs. 18.83%; *P* = 0.04). The types of ANCA were also different between the two diseases. The study highlighted that patients with IE more often developed c-ANCA, whereas those with HBV had both c-ANCA and p-ANCA, with the proportion of p-ANCA being more common in patients with HBV. The implication of the presence of c-ANCA in IE remains unclear. However, the infectious process may induce the production of c-ANCA, possibly polyclonal B-cell activation stimulated by bacterial unmethlylated oligodeoxynucleotides via Toll-like receptor 9 [30]. Jennette *et al.* reported that the release of neutrophil extracellular traps was involved in initiating the ANCA-associated autoimmune response, which was associated with a high titer of c-ANCA with anti-PR3 specificity [31]. Compared with IE, the pathogenesis of ANCA positivity in patients with HBV is thought to be a little different. It has been suggested that persistent antigen load secondary to chronic infection and a high viral replication rate lead to excessive formation of immunocomplexes containing cellular and/or humoral immunity, and these circulating immunocomplexes, deposited in intermediate- and small-sized vessels, initiate vasculitic processes [16, 32]. Vasculitic lesion development in HBV is believed to be associated with the HBeAg antigen or viral replication [33]. The majority of patients with IE and HBV were males; however, it was unclear whether a sex predilection for ANCA positivity exists in the two diseases. Previous case reports have described that more ANCA-positive patients with IE were found to be males [5]. IE occurs in 30% to 60% of patients with *Staphylococcus aureus* bacteremia and carries a mortality rate of 40–50% [34]. HBV is a chronic disease, and the mortality rate increases only when liver failure occurs [35]. The results showed that there was no direct relation between the deaths of the patients with IE and ANCA positivity (including 10 ANCA-positive and 27 ANCA-negative, 10/73 vs. 27/201; *P* = 0.96). Four patients with HBV-related PAN died of other causes after clinical recovery, including pancreatitis, cerebral bleeding, and sudden death [18].

**Table 1:** Characteristics and outcomes in patients with infective endocarditis and patients with hepatitis B virus with and without antineutrophil cytoplasmic antibodies among all previously reported cohort studies

	Patients with IE (n = 274)	Patients with HBV (n = 223)	*P* value
Mean age (yr)^a^	58.55	49.1	
Males/females^a^	135/64	132/91	0.07
ANCA-positive/ANCA-negative	73/201	42/181	0.04
ANCA (by IIF)			
c-ANCA	56	18	0.08
p-ANCA	14	21	0.01
c-ANCA + p-ANCA	─	1	0.79
ANCA (by ELISA)^b^			
PR3	34	19	0.9
MPO	7	8	0.21
PR3 + MPO	4	─	0.33
Outcomes			
Death ^c^	37 (ANCA-positive 10)	4	< 0.001

*Abbreviations: ANCA* Antineutrophil cytoplasmic antibodies, *c-ANCA* Cytoplasmic antineutrophil cytoplasmic antibodies, *ELISA* Enzyme-linked immunosorbent assay, *HBV* Hepatitis B virus, *IE* Infective endocarditis, *IIF* Indirect immunofluorescence, *MPO* Myeloperoxidase, *p-ANCA* Perinuclear antineutrophil cytoplasmic antibodies, *PR3* Proteinase 3

^a^Only three references have mentioned age and sex in patients with IE among all five references

^b^PR3- and MPO-ANCAs were detected by ELISA in patients with HBV only in two references

^c^Only four references describing patients with IE and two references describing patients with HBV have mentioned outcomes

## Conclusions

The findings in this study update and expand current understanding of ANCA positivity in patients with IE and HBV. Although PR3-ANCA and MPO-ANCA are serological markers for AAV, the interpretation of PR3-ANCA and/or MPO-ANCA positivity should consider the possibility of protracted infection. Identification of the causative pathogen is very important for successful treatment of IE. However, blood cultures can sometimes be negative because of preceding antibiotic treatment or pathogens difficult to detect with common culture methods. In addition, HBV-related vasculitis should be considered, especially HBV-related PAN, in a patient complicated with HBV infection. There are a variety of treatments, including surgery, antibiotics, corticosteroids and/or cyclophosphamide, antiviral agents, and even plasma exchange. As a result, how to choose the appropriate treatment will be challenging when several diseases present together, because if a patient with IE is administered a high dose of corticosteroids and/or cyclophosphamide, the consequences can be devastating. Therefore, renal biopsy is suggested if the patient’s condition allows. ANCA testing should be repeated after specific effective therapy, because some cases might require more aggressive treatment.

## Data Availability

All data generated or analyzed during this study are included in this published article.

## Abbreviations

AAV: Antineutrophil cytoplasmic antibody–associated vasculitis
ANCA: Antineutrophil cytoplasmic antibodies
c-ANCA: Cytoplasmic antineutrophil cytoplasmic antibodies
ELISA: Enzyme-linked immunosorbent assay
GN: Glomerulonephritis
HBeAg: Hepatitis B e-antigen
HBV: Hepatitis B virus
HCV: Hepatitis C virus
HIV: Human immunodeficiency virus
IE: Infective endocarditis
IIF: Indirect immunofluorescence
MPO: Myeloperoxidase
PAN: Polyarteritis nodosa
p-ANCA: Perinuclear antineutrophil cytoplasmic antibodies
SLE: Systemic lupus erythematosus
